# Methotrexate restores effector T cell responsiveness in juvenile idiopathic arthritis

**DOI:** 10.1186/1546-0096-9-S1-P131

**Published:** 2011-09-14

**Authors:** Maja Bulatović, Sebastiaan Vastert, Frederik Verweij, Nico Wulffraat, Femke van Wijk, Berent Prakken

**Affiliations:** 1 Department of Pediatric Immunology, University Medical Centre Utrecht, Wilhelmina Children’s Hospital, Netherlands

## Background

In juvenile idiopathic arthritis (JIA) the balance between immune activation and regulation is disturbed. Methotrexate (MTX) is a commonly used drug to induce disease remission in JIA patients, but the exact mechanisms of action remains unknown.

## Aim

We hypothesized that MTX induces remission by restoring the balance between effector (Teff) and regulatory T cells (Tregs). Therefore we examined the effects of MTX on Teff/Treg activation and function in JIA patients.

## Methods

Peripheral blood mononuclear cells (PBMCs) of 25 JIA patients were isolated prior to the start of MTX (T0) and 3 (T3) and 6 (T6) months after MTX start. Frequency and phenotype of FoxP3+ Tregs were analyzed *ex vivo* by flow cytometry and their suppressive function in CFSE suppression assays. Proliferation of CD4+ and CD8+ Teffs was determined with CFSE upon a 4-day culture in the presence of anti-CD3. Teffs cytokine production was measured *ex vivo* by flow cytometry upon PMA/ionomycin stimulation and in culture supernatants with luminex.

## Results

MTX does not induce changes in frequency, phenotype or suppressive capacity in Tregs at all time points. However, Teffs show significantly increased proliferation at T6 (mean: 76.2% of CD4+ T cells, SD 18.3%) than at T3 (39.8%, 18.7%) and T0 (49.0%, 21.1%) (p<0.01). Moreover, Teff proliferation at T6 is similar to healthy controls. Cytokine production (IL-10, IL-13, IL-17, IFNγ, TNFα) in supernatants was also increased at T6 compared to T3 and T0.

## Conclusion

In contrast to our hypothesis, we observed no effects of MTX on Treg. Surprisingly, clinical improvement in JIA patients treated with MTX was associated with increased proliferation and responsiveness of Teffs. This suggests clear modulatory effects of MTX on Teffs rather than immunosuppression.

